# Sensitivity and specificity of cerebrospinal fluid placental alkaline phosphatase in germinoma: An 8 pg/mL threshold

**DOI:** 10.1093/noajnl/vdag145

**Published:** 2026-06-05

**Authors:** Kentaro Chiba, Yasuo Aihara, Yuichi Oda, Kenta Masui, Takashi Komori, Takakazu Kawamata

**Affiliations:** Department of Neurosurgery, Tokyo Women’s Medical University, Tokyo, Japan; Department of Neurosurgery, Tokyo Women’s Medical University, Tokyo, Japan; Department of Neurosurgery, Tokyo Women’s Medical University, Tokyo, Japan; Department of Pathology, Graduate School of Biomedical Science, Nagasaki University, Nagasaki, Japan; Department of Neurosurgery, Tokyo Women’s Medical University, Tokyo, Japan; Department of Laboratory Medicine and Pathology, Tokyo Metropolitan Neurological Hospital, Tokyo, Japan; Department of Neurosurgery, Tokyo Women’s Medical University, Tokyo, Japan

**Keywords:** 8 pg/mL, cerebrospinal fluid placental alkaline phosphatase, clinical germinoma, false positive, true negative

## Abstract

**Background:**

Cerebrospinal fluid placental alkaline phosphatase (CSF-PLAP) is useful in diagnosing intracranial germinomas. However, reports on CSF-PLAP in histological subtypes other than germinoma remain limited. The primary aim of this study was to retrospectively analyze cases with histological diagnoses in which CSF-PLAP levels were measured and evaluate the sensitivity and specificity of this test using the clinically applied cutoff value of 8 pg/mL.

**Methods:**

We retrospectively analyzed 134 patients with intracranial disease who underwent CSF-PLAP testing for diagnostic purposes and had confirmed histopathological diagnoses without receiving any adjuvant therapy before, or between CSF-PLAP measurement and surgery. The cases were categorized as true positive (TP), true negative (TN), false positive (FP), false negative, or clinical germinoma. Diagnostic accuracy was evaluated based on sensitivity, specificity, and statistical comparisons between groups.

**Results:**

Among the 134 analyzed cases, 30 were classified as TP, 99 as TN, and 5 as FP. Using the 8 pg/mL cutoff, CSF-PLAP testing demonstrated a sensitivity of 100% and a specificity of 95.2%. The mean CSF-PLAP level was significantly higher in the TP group than in the FP group, and the TP group participants were significantly younger than the FP group participants. No significant differences in CSF-PLAP levels or age were found between the TP and clinical germinoma groups, supporting their clinical equivalence.

**Conclusions:**

CSF-PLAP using an 8 pg/mL cutoff demonstrates high diagnostic accuracy for intracranial germinoma. Recognition of potential false-positive results and consideration of sampling site may further improve clinical interpretation of CSF-PLAP measurements.

Key Points

- CSF-PLAP shows high diagnostic accuracy for germinoma using a clinically applied 8 pg/mL cutoff.
- PLAP levels were evaluated in diverse CNS disorders beyond germinoma.
- False-positive CSF-PLAP results highlight the need for careful interpretation of elevated CSF-PLAP values in clinical diagnosis.

Importance of the Study

Noninvasive biomarkers are increasingly valuable in diagnosing central nervous system tumors, where surgical procedures carry significant risks. Previous studies of cerebrospinal fluid placental alkaline phosphatase (CSF-PLAP) have largely focused on germinoma, with limited evaluation of PLAP levels in other intracranial diseases. This study addresses that gap by systematically describing non-germinomatous disorders in which CSF-PLAP was measured, thereby clarifying disease spectra associated with true-negative and false-positive results. Using the clinically implemented cutoff corresponding to the assay detection limit (8 pg/mL), we demonstrated high sensitivity (100%) and high specificity (95.2%) for intracranial germinoma. With increasing case accumulation, false-positive CSF-PLAP results were identified, highlighting the importance of recognizing such findings in clinical interpretation. Serial CSF-PLAP measurements in selected patients further suggested utility for treatment monitoring. Overall, validation of the currently used 8 pg/mL cutoff supports CSF-PLAP as a robust biomarker that can reduce diagnostic delays and help avoid high-risk surgical biopsy in suspected germinoma.

## Introduction

The usefulness of measuring placental alkaline phosphatase in the cerebrospinal fluid (CSF-PLAP) for diagnosing germinoma of the central nervous system has been well documented.^1-7^ Over time, the cutoff value used to determine diagnostic positivity has evolved; it was initially reported at 30 picograms per milliliter (pg/mL) by Watanabe et al and later adjusted to 10 pg/mL by Aihara et al.^4^^,^^5^ At present, a cutoff value of 8 pg/mL—corresponding to the assay detection limit—is currently applied in clinical practice in Japan for the diagnosis of intracranial germinoma.^2^^,^^3^

Systematic evaluations of CSF-PLAP levels across alternative intracranial pathologies—including non-germinomatous primary brain tumors, metastatic brain tumors, and neurodegenerative disorders—remain scarce. In this article, we conducted a retrospective analysis of 134 patients with newly diagnosed, untreated intracranial lesions who underwent CSF-PLAP measurement, with particular emphasis on correlating PLAP levels with detailed histopathological diagnoses across both germinomatous and non-germinomatous disease entities. Applying the clinically implemented threshold of 8 pg/mL, cases were stratified into true positive (TP), true negative (TN), false positive (FP), false negative (FN), and clinical germinoma categories, and diagnostic performance was evaluated in terms of sensitivity and specificity. Through this approach, we sought to clarify the diagnostic utility and limitations of CSF-PLAP in contemporary clinical practice.

## Methods

### Inclusion and Exclusion Criteria

This study was approved by our institutional ethics committee (Approval No. 2024-0157) and performed in accordance with the Declaration of Helsinki. Due to its retrospective design, the institutional review board waived the requirement for informed consent. All identifiers were removed from our records after analyses to ensure privacy. Patients were eligible for inclusion if they underwent CSF-PLAP measurement for diagnostic purposes, had histopathological confirmation, and subsequently received treatment at our institution between 2006 and 2024.

PLAP has two isoforms (Regan and Nagao).^8^ We used monoclonal antibodies prepared against purified human PLAP (MoAb24), which react with both isoforms.^5^ At present, CSF-PLAP testing in Japan is available through external clinical laboratories (e.g., SRL, Inc., Tokyo, Japan). CSF samples were collected through lumbar puncture, directly from the cerebral ventricles in cases involving the treatment of hydrocephalus, or both in selected cases. In cases where both lumbar and ventricular CSF samples were obtained simultaneously, the higher of the two measured values was adopted for the primary diagnostic analysis. In addition, lumbar and ventricular CSF samples were analyzed separately to evaluate potential concentration gradients along the neuraxis. For lumbar and ventricular sampling, at least 2 mL of CSF was obtained for each case, and PLAP levels were measured in accordance with previously reported methods.^4^^,^^5^ When collecting the CSF from the ventricles, drainage tubing with saline prevented sample dilution.

CSF-PLAP measurements were performed for diagnostic purposes prior to any therapeutic intervention. In patients who underwent multiple CSF-PLAP measurements during the course of treatment, only the value obtained at the time of diagnosis was included in the primary statistical analyses. This approach was adopted to ensure consistency across cases and to avoid bias related to treatment-induced changes in CSF-PLAP levels.

In addition to the diagnostic-time measurements used for statistical evaluation, a subset of patients clinically diagnosed with germinoma underwent serial CSF-PLAP measurements at predefined time points during their clinical course. These longitudinal measurements were not included in the primary statistical analyses but were recorded for descriptive evaluation of temporal changes in CSF-PLAP levels in relation to treatment.

Only cases with histopathological confirmation were included; those without histological diagnosis were excluded, even when strong supportive clinical evidence was present, including highly specific tumor markers or antibodies in serum or CSF, characteristic radiological findings on CT, SPECT, or MRI, or intraoperative observations.

Furthermore, we excluded cases in which patients had undergone treatments prior to CSF-PLAP measurement that might have altered tumor burden and thereby lead to confounding results, including debulking surgery (excluding biopsy), radiotherapy, chemotherapy, or corticosteroid administration, as well as cases in which CSF-PLAP levels were measured before therapy but radiotherapy or chemotherapy had been administered before histopathological confirmation, since such interventions could eradicate germinoma components. Cases with recurrence or those previously treated at other institutions were excluded in accordance with the aforementioned criteria. Among the latter group, non-germinomatous germ cell tumors (NGGCTs), including mature teratomas, immature teratomas, yolk sac tumors, embryonal carcinomas, choriocarcinomas, and mixed germ cell tumors containing NGGCT and germinoma components are of particular importance. Because NGGCTs are frequently managed with upfront chemotherapy or radiotherapy, the germinoma components—which are highly sensitive to these interventions—may have already disappeared by the time of resection, making accurate assessment of their presence or absence difficult. For this reason, such cases were excluded from the present evaluation. No pregnant patients were included in the study. A limited number of CT scans was considered permissible.

### Definitions

In this study, cases were classified into diagnostic categories based on the CSF-PLAP concentration and the presence or absence of germinoma components from histopathological examination. In principle, histopathological diagnoses were rendered by a board-certified neuropathologist in accordance with the 2021 World Health Organization classification; nevertheless, for certain older cases, the original diagnoses based on the contemporaneous WHO classification were retained.^9^

A case was defined as a TP when the CSF-PLAP concentration was equal to or greater than 8.0 pg/mL and the presence of germinoma components was histologically confirmed. Conversely, a case was considered a TN when the CSF-PLAP concentration was less than 8.0 pg/mL and histopathology confirmed either a non-germinoma tumor or a tumor not containing any germinoma components.

A case was categorized as a FP when the CSF-PLAP level was 8.0 pg/mL or higher, but the histological diagnosis revealed a tumor other than a germinoma. In contrast, a FN was defined as a case in which the CSF-PLAP concentration was less than 8.0 pg/mL and histological analysis demonstrated the presence of germinoma components. In addition, among the cases excluded due to the absence of histopathological confirmation, those exhibiting elevated CSF-PLAP levels and demonstrating treatment responses to radiotherapy and/or chemotherapy consistent with the clinical course of germinoma were separately categorized as the “clinical germinoma” group.

### Statistical Analysis

Statistical analysis was retrospectively performed using JMP Pro software for Windows, version 18 (SAS Institute Inc., Cary, NC, USA). To assess the statistical significance of the differences among these categories and the clinical germinoma groups, CSF-PLAP concentrations and patient age were compared. These comparisons were performed using the Mann-Whitney U-test, which is suitable for samples with unequal variance. Statistical significance was set at *P* < .05.

## Results

**Figure 1.**
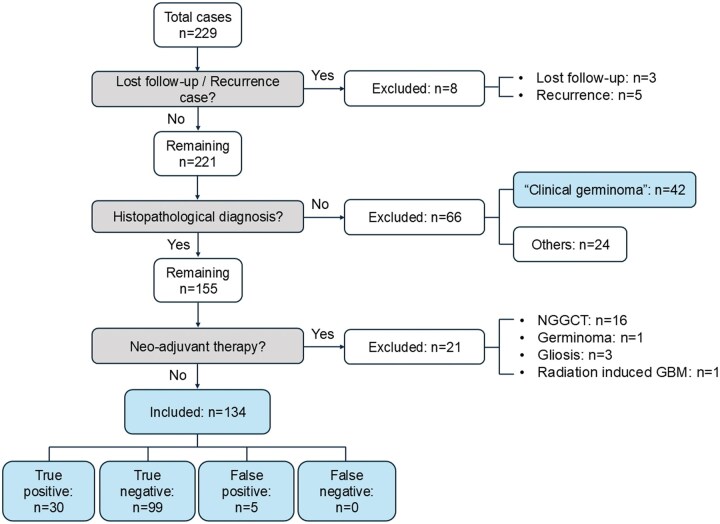
Patient inclusion and exclusion flowchart. A total of 229 patients underwent CSF-PLAP testing, of whom 134 were included in the final analysis. Of the 229 patients, 95 were excluded due to incomplete clinical data (*n* = 3), recurrence (*n* = 5), lack of histopathological diagnosis (*n* = 66), or prior treatment affecting tumor burden (*n* = 21). Abbreviations: NGGCT, non-germinomatous germ cell tumor, GBM, glioblastoma multiforme.

During the study period, 384 CSF-PLAP tests were performed on 229 patients. After applying the exclusion criteria, 95 cases were excluded from the analysis. Finally, 134 cases were included in the study cohort, each corresponding to one diagnostic phase (Figure 1). CSF samples were obtained via lumbar puncture only in 89 cases and from the ventricular system only in 37 cases. In addition, paired lumbar and ventricular CSF samples were available in 8 cases. The average age at the time of CSF-PLAP measurement among the included patients was 26.4 years, with a 95% confidence interval (CI) of 23.3 to 29.4 years (range, 0-79 years). The cohort comprised 80 men and 54 women.

**Figure 2.**
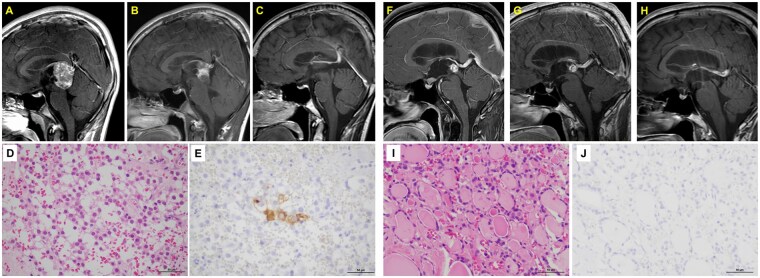
Representative case of mixed germ cell tumor (immature teratoma with germinomatous component) of an 11-year-old boy, who presented a germinoma component with elevated CSF-PLAP value at 10 pg/mL. (A) MRI of T1 weighted image (WI) with contrast-enhanced matter at initial diagnosis. CSF-PLAP showed 467 pg/mL. (B) MRI T1WI with contrast-enhanced matter after one course of ICE treatment and just before resection surgery. CSF-PLAP revealed 10.0 pg/mL at this point. (C) The latest MRI, 7 years after the initial diagnosis, revealed no recurrence and CSF-PLAP was within 8.0 pg/mL. Histopathological findings revealed typical two-cell patterns on HE stains (D) and positive for PLAP (E) and c-kit, indicating the presence of a germinoma component. Another representative case of a mixed germ cell tumor (mature teratoma with a germinomatous component) in a 33-year-old man. (F) Contrast-enhanced T1-weighted MRI at initial diagnosis, showing an enhancing lesion. The CSF-PLAP level was 571 pg/mL. (G) Contrast-enhanced T1-weighted MRI obtained after two course of ICE chemotherapy and immediately before surgical resection. At this time point, the CSF-PLAP level had decreased to 8.0 pg/mL. (H) Follow-up MRI obtained 8 years after the initial diagnosis revealed no evidence of tumor recurrence, and CSF-PLAP levels remained below 8 pg/mL. Histopathological examination demonstrated teratomatous elements on hematoxylin and eosin staining (I) and was negative for PLAP (J) and c-kit immunostaining, indicating the absence of residual germinoma components. Scale bar = 50 μm. Abbreviations: CSF-PLAP, cerebrospinal fluid placental alkaline phosphatase.

To illustrate the relationship between CSF-PLAP levels and histopathological findings, representative cases were examined. In one case, a CSF-PLAP level of 10 pg/mL was observed immediately prior to tumor resection, and histopathological examination confirmed the presence of germinoma components (Figure 2A-E). In contrast, another case showed a CSF-PLAP level below the detection limit of 8 pg/mL prior to resection, and histopathology revealed no germinoma components, with the tumor diagnosed as a teratoma (Figure 2F-J). Consistent with these representative findings, germinoma components were identified in all cases with CSF-PLAP levels at or above the detection limit of 8 pg/mL at the time of resection in the present cohort, including cases with values only marginally exceeding the cutoff, whereas no germinoma components were identified in cases with CSF-PLAP levels below the detection limit.

Among the 134 cases, 8 had paired CSF samples obtained from both the ventricular system and the lumbar compartment. Lumbar CSF-PLAP levels were generally higher than ventricular CSF-PLAP levels, suggesting the presence of a rostrocaudal gradient of PLAP concentration within the cerebrospinal fluid. Ventricular and lumbar PLAP levels showed a strong positive correlation (Spearman’s *ρ*  =  0.875, *P* = .004).

When paired CSF samples were available, the higher PLAP value was used for statistical analysis, as described in the Methods section. At the threshold of 8.0 pg/mL, the test achieved a sensitivity of 100% (95% CI: 88.4%-100%) and a specificity of 95.2% (95% CI: 89.1%-98.4%), enabling accurate identification of TP cases without missing any germinoma components, while maintaining a very low FP rate. These findings support the clinical utility of using a CSF-PLAP cutoff of approximately 8.0 pg/mL for accurate diagnosis of germinomas. For reference, when the lower PLAP value was adopted in cases with paired CSF sampling, two cases were classified as false positives. Both cases were PLAP-positive in lumbar CSF, with lumbar PLAP values of 9.4 pg/mL and 29.5 pg/mL, respectively. Under these conditions, the recalculated sensitivity and specificity were 95.6% (95% CI: 85.2%-98.8%), and 96.5% (95% CI: 91.4%-98.6%), respectively, at a cutoff of 8.0 pg/mL.

Furthermore, when lumbar CSF samples alone were analyzed, the sensitivity and specificity of PLAP for the diagnosis of germinoma were 100.0% (95% CI, 66.4%-100.0%) and 94.4% (95% CI, 81.3%-98.6%), respectively. When ventricular CSF samples alone were analyzed, the sensitivity and specificity were 100.0% (95% CI, 29.2%-100.0%) and 75.5% (95% CI, 65.9%-83.1%), respectively.

**Figure 3.**
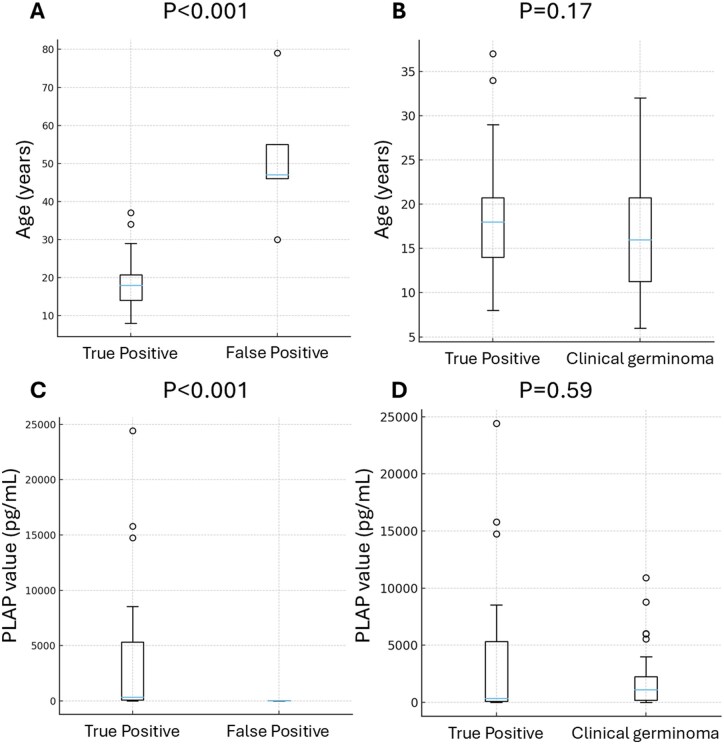
Statistical analysis of PLAP levels between TP versus FP and clinical germinoma versus TP. (A, C) There was a statistically significant difference in age (<.001) and in PLAP value (*P* < .001) between the TP group and the FP group. (B, D) There was no significant difference in age (*P* = .17) or in PLAP value (*P* = .59) between the TP group and the clinical germinoma group, indicating that both groups were clinically comparable. Abbreviations: PLAP, placental alkaline phosphatase; FP, false positive; TP, true positive.

In the primary analysis, 30 cases were classified as TP, accounting for 22.3% of the total cases. These patients had a mean age of 19.1 years (95% CI: 16.6-21.5 years; range, 8-37 years) and included 25 men and 5 women. The TN group comprised 99 patients, representing 73.3% of the total, with a mean age of 26.8 years (95% CI: 23.1-30.6 years; range, 0.3-74 years), with 52 men and 47 women. Five cases (4.4% of the total) were identified as FP; these patients had a mean age of 51.4 years (95% CI: 29.2-73.6 years; range, 30-79 years), with 3 men and 2 women. No case of FN was identified in this study. The FP group had a significantly higher mean age compared with the TP group (*P* < .001) (Figure 3A). There were no statistically significant differences in age between the TP group and the TN group (*P* = .21).

In addition to TP cases, 42 patients were clinically diagnosed with germinoma based on elevated CSF-PLAP levels without histological confirmation and were classified into the clinical germinoma group with a mean age of 16.7 years (95% CI: 14.5-18.9 years; range, 6-32 years), comprising 32 men and 10 women. There was no statistically significant difference in age between the TP group and the clinical germinoma group (*P* = .17; Figure 3B).

The mean CSF-PLAP concentration in the TP group was 3,494.4 pg/mL (95% CI: 1304.7-5684.1 pg/mL). In comparison, the FP group had a significantly lower mean CSF-PLAP value of 19.7 pg/mL (95% CI: 7.3-32.2 pg/mL; *P* < .001; Figure 3C).

The clinical germinoma group showed a mean CSF-PLAP level of 1839.8 pg/mL (95% CI: 1083.4-2596.2 pg/mL). There was no statistically significant difference in CSF-PLAP levels (*P* = .59) between the TP group, indicating that they were clinically comparable (Figure 3D).

### Serial CSF-PLAP Measurements during Treatment

**Figure 4.**
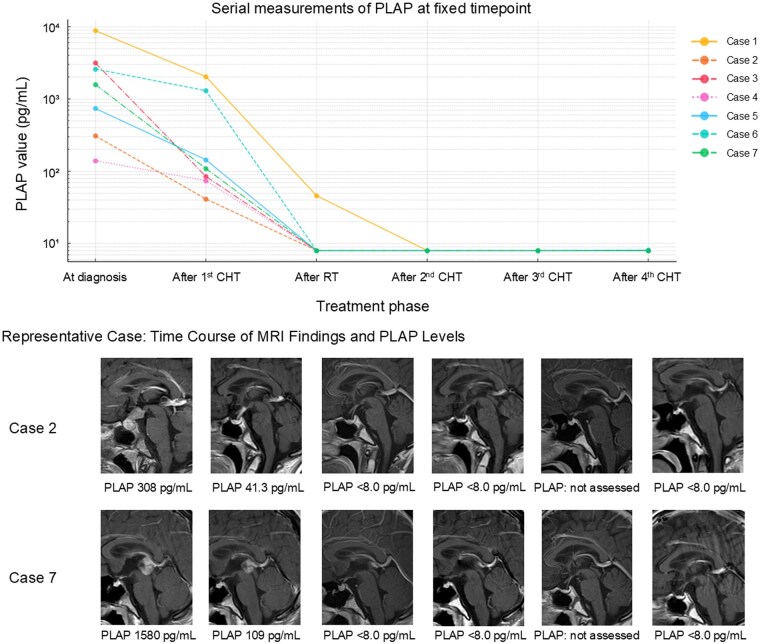
Serial measurement of CSF-PLAP at a fixed time point. Abbreviations: CHT, chemotherapy, CSF-PLAP, cerebrospinal fluid placental alkaline phosphatase RT, radiotherapy.

Among the 42 patients clinically diagnosed with germinoma, seven underwent serial CSF-PLAP measurements at predefined time points during their clinical course. In most of these patients, CSF-PLAP levels progressively decreased during treatment and became undetectable after completion of radiotherapy. Representative longitudinal changes in CSF-PLAP levels are shown in Figure 4.

Among the five FP cases, histological diagnoses included two cases of metastatic brain tumors, one case of granulomatosis with polyangiitis (GPA), one case of diffuse large B-cell lymphoma (DLBCL), and one case of pineoblastoma (Table 1). There were 99 cases in true negative group (Table 2). Glioma was the most frequent diagnosis, with 36 cases, followed by craniopharyngioma and other tumors that preferably arise in the same region as germinoma. A total of 21 cases of NGGCT with documented CSF-PLAP measurements were identified. Among these, 16 cases were excluded, all on the grounds of having received prior chemotherapy or radiotherapy. The histological distribution of the excluded cohort was as follows: mature teratoma (*n* = 6), immature teratoma (*n* = 6), embryonal carcinoma (*n* = 1), yolk sac tumor (*n* = 2), and choriocarcinoma (*n* = 1). As these 16 cases were excluded, they were not considered in the present analysis. In the remaining five cases, CSF-PLAP and/or other tumor markers at initial presentation were either within the normal range or only marginally elevated, and therefore prior chemotherapy or radiotherapy was deemed unlikely to have been efficacious. Accordingly, following acquisition of tumor marker data, prompt craniotomy and tumor resection were performed.

**Table 1.** List of five cases of histopathological diagnosis in the false positive group

Diagnosis	Number of cases	PLAP value (pg/mL)
Metastatic carcinoma	2	
Breast cancer	1	10.1
Lung cancer	1	14.9
Diffuse large B-cell lymphoma (DLBCL)	1	24.2
Granulomatosis with polyangiitis (GPA)	1	14.4
Pineoblastoma	1	35.1

**Table 2.** List of histopathological diagnoses of the true negative group

Diagnosis	Number of cases
Glioma	36
Pilocytic astrocytoma	11
Pleomorphic xanthoastrocytoma	1
Ganglioglioma	2
Diffuse astrocytoma	3
Anaplastic astrocytoma	1
Diffuse midline glioma, H3K27M altered	10
Glioblastoma	6
NOS	2
Craniopharyngioma	10
Adamantinomatous type	6
Papillary type	3
Not defined	1
Medulloblastoma	7
Non-germinomatous germ cell tumor (NGGCT), mature teratoma	3
Mature teratoma	2
Immature teratoma	1
Pineal parenchymal tumor of intermediate differentiation (PPTID)	6
Grade 2	2
Grade 3	4
Diffuse large B-cell lymphoma (DLBCL)	3
Pineoblastoma	3
PitNET/adenoma	3
Meningioma	2
Lymphocytic hypophysitis	2
Cavernoma	2
MOG antibody-associated disease	2
Pineocytoma	2
Papillary tumor at pineal region (PTPR)	2
Rosette forming glioneuronal tumor (RGNT)	2
Peripheral primitive neuroectodermal tumor (p-PNET)	1
Pituicytoma	1
Primitive neuroectodermal tumor (PNET) (old classification)	1
Supra-tentorial ependymoma	1
Arachnoid cyst	1
Lhermitte-Duclos disease	1
Metastatic carcinoma (breast cancer)	1
Langerhans cell histiocytosis (LCH)	1
Inflammatory change	1
Epidermoid	1
Colloid cyst	1
Chordoid glioma	1
Gliosis	1
Central neurocytoma	1

## Discussion

The cutoff value for CSF-PLAP evolved from 30 pg/mL to 10 pg/mL.^4^^,^^5^ In this retrospective study, a cutoff of 8.0 pg/mL resulted in a sensitivity of 100% and a specificity of 95.2%. This indicates that CSF-PLAP testing with an 8.0 pg/mL cutoff can detect the presence of germinoma components with very high sensitivity and high specificity.^4^ No false-negative cases were observed in this cohort.

Germinoma components are highly responsive to chemotherapy and radiotherapy, and a cure can occasionally be achieved without surgery.^4^^,^^10-15^ Moreover, in cases of mixed germ cell tumors containing germinoma and NGGCT components, administering chemotherapy or radiotherapy before surgery can reduce the tumor volume of the germinoma portion, which can help lower surgical difficulty and assist in determining surgical timing.^2^^,^^3^ Thus, CSF-PLAP testing provides important clinical value not only by offering a noninvasive alternative to diagnostic surgery but also by supporting evidence-based treatment planning and risk classification.^3^^,^^4^

Reflecting these benefits, in recent years, we have increasingly encountered cases where treatment has been initiated without histological diagnosis based solely on elevated CSF-PLAP levels. These cases were categorized into the clinical germinoma group in this article. Our analysis revealed no statistically significant differences in CSF-PLAP levels or patient age between the clinical germinoma and TP groups, indicating their equivalence from a clinical perspective. Furthermore, serial CSF-PLAP measurements in a subset of clinically diagnosed germinoma cases demonstrated that marker levels decreased in parallel with treatment and became undetectable in most patients following combined chemotherapy and radiotherapy. Although limited in number, these observations suggest that serial CSF-PLAP assessment may provide a noninvasive means of monitoring tumor dynamics during treatment.

However, with more cases, FPs emerged. Five cases of FPs were identified in this study. Washiyama et al examined various brain tumor types, not limited to germ cell tumors, using PLAP immunohistochemistry and not CSF-PLAP and reported similar findings.^8^ Among these, two metastatic brain tumors were positive for PLAP.^8^ Alkaline phosphatase comprises distinct isoenzymes, including the Regan-type and Nagao-type.^4^^,^^8^ Washiyama et al speculated that FPs may arise from the cross-reactivity between these two PLAP subtypes or from certain cancers that produce PLAP.^8^ In our study, as similarly reported by Washiyama et al two metastatic brain tumors were in the FP group, one originating from lung cancer and the other from breast cancer. Simultaneously, we encountered metastatic brain tumors without elevated CSF-PLAP levels, suggesting that the variation in PLAP expression depends on the primary site or tumor histology; however, our sample size was too small to conclude.

Additionally, our FP group included one case each of pineoblastoma, DLBCL, and GPA, none reported in the study by Washiyama et al.^8^ GPA is exceedingly rare, and it remains unclear whether the FP is disease-specific; further investigation is needed. None of the three other pineoblastoma cases at our institution showed elevated CSF-PLAP levels. Similarly, DLBCL cases in our cohort were generally negative for CSF-PLAP. The pineoblastoma case in the FP group showed histologically characteristic features but was somewhat atypical as the patient was 30 years old, slightly older than the usual epidemiological profile. Whether these FP findings in pineoblastoma and DLBCL represent case-specific or disease-related patterns requires further case accumulation, similar to GPA. Another possible explanation for the FP results is blood contamination in CSF samples, which can elevate CSF-PLAP levels. Although grossly blood-contaminated samples were avoided whenever possible, minor blood contamination cannot be completely excluded in retrospective analyses. Therefore, ensuring the integrity and cleanliness of CSF samples, such as by avoiding blood admixtures, is essential for obtaining accurate test results.

While investigating the causes of FPs is important for improving the specificity of CSF-PLAP testing, our statistical analysis revealed that the FP group had significantly lower CSF-PLAP values and was older than the TP group. These findings suggest that in cases where CSF-PLAP levels are relatively low (e.g., below approximately 30 pg/mL) and the patient is older than the typical age profile for germ cell tumors (i.e., 40 years or older), clinicians should consider the possibility of alternative diagnoses, even if CSF-PLAP is elevated in cases of atypical neuroimaging, blood test findings, or response to adjuvant therapy.^1^^,^^13^^,^^14^^,^^16^

When specificity was prioritized, setting the cutoff value at 31.0 pg/mL resulted in an area under the receiver operating characteristic curve of 0.998, yielding a high specificity of 99.0% while maintaining a sensitivity of 96.7%. Nevertheless, because the principal objective of PLAP measurement is to detect germinoma components without any false negatives, we consider that a cutoff value of 8.0 pg/mL, which ensures 100% sensitivity, represents the optimal threshold.

In contrast, a negative CSF-PLAP result should prompt the consideration of diseases other than germinoma or NGGCTs lacking germinoma components.^3^ MRI findings alone are often insufficient for a definitive diagnosis.^17^ In such cases, further evaluation using disease-specific antibodies or imaging studies is necessary, and surgical biopsy should be considered when appropriate. For example, while bifocal lesions are a characteristic imaging feature of germinomas, we reported a case in which a patient with bifocal lesions had negative CSF-PLAP and was ultimately diagnosed with a pineal parenchymal tumor of intermediate differentiation.^14^^,^^18-20^

Although no false-negative cases were identified in this article, false-negative results have been reported previously, and additional cases may emerge as CSF-PLAP testing is applied more broadly in clinical practice.^21^

Furthermore, avoiding invasive biopsy procedures and proceeding directly to treatment without delay provides substantial clinical value.^20^ Nevertheless, lumbar puncture carries certain risks, such as postural headache due to CSF depletion, which should not be overlooked. In addition, higher CSF-PLAP levels were observed in samples obtained by lumbar puncture compared with those obtained from ventricular CSF sampling. The mechanism underlying this concentration gradient remains unclear, and given the limited number of cases analyzed (*n* = 8), further evaluation in larger cohorts is warranted. These findings suggest that lumbar CSF sampling may be more appropriate for diagnostic PLAP measurement when feasible. However, lumbar puncture is contraindicated in patients with hydrocephalus. Therefore, ventricular CSF sampling may be required in such cases, whereas lumbar CSF sampling may be considered when hydrocephalus has resolved.

Finally, although it has been excluded and is not shown in the present manuscript, our experience with a case of recurrent germinoma in which PLAP elevation was observed at relapse, followed by PLAP normalization after treatment and sustained remission thereafter, suggests that PLAP measurement helps not only to monitor treatment response during initial therapy but also to detect recurrence.^2^

This study has several limitations. Its retrospective design and diagnostic-driven case selection may have introduced selection bias, as CSF-PLAP measurements were primarily performed in patients with intracranial lesions suspected of germinoma, which typically arise in the suprasellar or pineal regions; consequently, the disease spectrum of the true-negative group may not fully represent all intracranial pathologies. In addition, non-germinomatous germ cell tumors were excluded because upfront treatment or surgical intervention may eliminate germinoma components prior to histopathological confirmation, further limiting generalizability.

In conclusion, CSF-PLAP measurement was found to be useful in diagnosing germinoma. By setting the cutoff value at 8.0 pg/mL, diagnosing germinomas with a sensitivity of 100% and specificity of 95.2% was possible. The ability to assess the presence or absence of germinoma components, which are highly sensitive to chemotherapy and radiotherapy, using a minimally invasive test rather than surgery has significant clinical value.

In addition, the present analysis suggests the presence of a rostrocaudal gradient in CSF-PLAP concentrations, with higher levels observed in lumbar samples compared with ventricular samples.

Although no cases of FN were observed in this study, several FP results were noted. Based on statistical analyses, these FP cases tended to have relatively low CSF-PLAP levels (<30 pg/mL) and occurred in patients whose ages deviated significantly from the typical age range for germ cell tumors. Therefore, even when CSF-PLAP results are positive, clinicians must be cautious and consider the possibility of other diseases, particularly in patients with atypically low PLAP values or those outside the usual age range for germinoma for diagnosis and treatment planning.

## Data Availability

The data supporting the findings of this study are available from the corresponding author upon reasonable request. De-identified individual-level data, including CSF-PLAP concentrations and clinical diagnoses, can be shared in accordance with institutional ethical guidelines.
